# The risk of malignancy of the thyroid nodule/focal lesion – an assessment by ultrasound, based on our own scoring system

**DOI:** 10.1186/1756-6614-6-S2-A1

**Published:** 2013-04-05

**Authors:** Zbigniew Adamczewski, Andrzej Lewiński

**Affiliations:** 1 Polish Mother’s Memorial Hospital - Research Institute, Lodz, Poland; 2 Department of Endocrinology and Metabolic Diseases, Medical University of Lodz, Poland

## 

Ultrasonography (US) is the most commonly performed test used to visualize the thyroid. This examination is safe and readily available, hence it is very often carried out. Ultrasound observations allow the description of structural abnormalities of the thyroid gland evaluated in whole, or of nodules/focal lesions present in the thyroid. An ultrasound examination measures the acoustic density of tissues. The image is displayed on the apparatus screen in grayscale (B-mode). An assessment of blood flow, showing the location and intensity of thyroid vascularity is a newer US technique that uses the Doppler effect for measurements. In turn, elastography is the latest application of US, i.e. the examination which is based on the assessment of the, so-called, stiffness of lesions in comparison with the stiffness of normal thyroid tissue (i.e., the tissue of morphologically normal appearance). A common performance of US of the neck region, often carried out because of the indications that are not related to the thyroid gland (e.g., evaluation of salivary glands, assessment of blood flows in carotid and vertebral arteries) leads to the disclosure of a number of lesions detected incidentally in the thyroid gland itself. In turn, it results in the need for proper interpretation of US images in order to optimize further diagnostic and therapeutic procedures. Previous studies on the use of US to determine the nature of focal lesions/thyroid nodules in terms of their benign vs. malignant character did not allowed to give a clear answer. These studies have not led to the general acceptance of cancer risk US patterns. Thus, at the present stage of knowledge, it is not possible to accept the categorical and definitive determination of the nature of lesions in terms of "malignant" vs. "benign" – on the basis of US image only. However, the studies have helped to determine the characteristics of a group of US image features, the presence of which is associated with a higher risk of cancer diagnosis. Endocrinologists diagnosing the patients with thyroid diseases are expecting to have exact guidelines and recommendations that would make easier to decide on the appropriate management. This applies in particular to answer the question: what is a greater risk to the patient - whether to undergo a surgical treatment, or abandon it? Analysis of data from the medical literature and our personal experience indicate the possibility of assigning certain patterns of US appearance to the risk of lesion malignancy. The multiplicity of possible combinations of co-occurrence of different US characteristics indicates that the most appropriate way of an objective assessment of the image is the introduction of an appropriate scoring system which reflects the accumulation of suspicious US features and the category of US risk of malignancy expressed by a number of assigned points. This is precisely our issue that we have decided to propose; our scale divides the patterns of US image risk into 3 levels: high, intermediate and low risk. In our scoring system, the finding of pathologically altered lymph nodes (usually enlarged) and/or a confirmation of the fast growing lesions/thyroid nodules (augmentation) is equal to granting 3 points for each of these characteristics. Hypoechogenicity, the presence of microcalcifications, shape of lesion - close to the rotational ellipsoid with the longest axis of a vertical (shape of an egg in upright position), as well as the presence of abnormal vascular flows (the increased and chaotic blood flows centrally located in the lesion, or totally absent blood flows in hypoechogenic lesions) is equal to the grant 1 point for each of the features. The absence of a regular thin "halo", solid structure (composition) of the lesion, the focus/nodule diameter of 3 cm and more and its uneven, blurred outlines (margins) are associated with granting 0.5 points for each of the features. The sum of granted points, resulting from the presence of US features specified above, is the basis of qualification for US risk levels. The score ranging from 0 to 4 points, awarded on the basis of thyroid US, speaks for a low risk of thyroid cancer, the score from 4 to 7 points – corresponds to intermediate risk, and the granting of 7 points or more qualifies to the group of US high-risk of malignancy. The use of this scoring system can help make the right clinical decisions, especially in patients with non-diagnostic smears (category I of The Bethesda System for Reporting Thyroid Cytopathology - TBSRTC), also in patients with the smears, in case of which it is impossible to determine the exact nature of the smear (category III TBSRTC, being also called "category of exclusion"), as well as in patients with benign lesions (category II TBSRTC) when such a cytological diagnosis seems unlikely in combination with the US image indicating a high risk of malignancy.

